# Stillbirths in Rural Hospitals in The Gambia: A Cross-Sectional Retrospective Study

**DOI:** 10.1155/2010/186867

**Published:** 2010-06-30

**Authors:** Abdou Jammeh, Siri Vangen, Johanne Sundby

**Affiliations:** ^1^Section for International Health, Department of General Practice and Community Medicine, Institute of Health and Society, University of Oslo, Blindern, 0317 Oslo, Norway; ^2^Oslo University Hospital, Department of Obstetrics and Gynaecology, National Resource Centre for Women's Health, N-0027 Oslo, Norway; ^3^Section for International Health, Department of General Practice and Community Medicine, Institute of Health and Society, University of Oslo, P.O. BOX 1130 Blindern, N-0318 Oslo, Norway

## Abstract

*Objective*. We determined the stillbirth rate and associated factors among women who delivered in rural hospitals in The Gambia. *Method*. A cross-sectional retrospective case review of all deliveries between July and December 2008 was undertaken. Maternity records were reviewed and abstracted of the mother's demographic characteristics, obstetric complications and foetal outcome. *Main Outcome Measure*: The stillbirth rate was calculated as deaths per 1000 births. *Results*. The hospital-based stillbirth rate was high, 156 (95% CI 138–174) per 1000 births. Of the 1,519 deliveries, there were 237 stillbirths of which 137 (57.8%) were fresh. Severe obstetric complication, birth weight *<*2500 g, caesarean section delivery, and referral from a peripheral health facility were highly significantly associated with higher stillbirth rates, OR = 6.68 (95% CI 3.84–11.62), 4.47 (95% CI 3.04–6.59), 4.35 (95% CI 2.46–7.69), and 3.82 (95% CI 2.24–6.51), respectively. Half (50%) of the women with stillbirths had no antenatal care OR = 4. 46(95% CI 0.84–23.43). *Conclusion*. We observed an unacceptably high stillbirth rate in this study. As most of the stillbirths were fresh, improved intrapartum care supported by emergency transport services and skilled personnel could positively impact on perinatal outcomes in rural hospitals in The Gambia.

## 1. Introduction

Stillbirths and neonatal deaths remain a huge challenge in the care of pregnant women, especially in developing countries [1]. Over 3.3 million stillbirths and more than 3 million early neonatal deaths occur every year. A vast majority (98%) takes place in developing countries where stillbirths represent over half of the perinatal deaths [2]. Complications during pregnancy and child birth are known to be closely associated with high stillbirth and perinatal mortality rate [3]. Perinatal mortality and stillbirth rates are important indicators of the quality of antenatal and obstetric care in a community [4]. Despite an important indicator stillbirths are invisible in global policy and programme priorities. They are usually not captured in local data collecting systems [2, 5]. Lack of a well-defined programme agenda, coupled with the lack of data, and social invisibility, deter action and investments for stillbirth prevention and reduction [2].

Being cognizant of the distribution of stillbirths (fresh and macerated) and deaths within the immediate postpartum period may help to detect shortcomings in the quality of antenatal and obstetric care given to the pregnant woman, hence prioritize appropriate intervention programmes [6]. Data on the frequency and distribution of these adverse births outcomes are important for planning maternal and child health services in developing countries [7]. As a drive to achieve the Millennium Development Goals (MDGs), the Government of The Gambia developed a national road map to accelerate the reduction of maternal and newborn morbidity and mortality [8]. However, this strategy remains a challenge due to weak health system, gross shortage of skilled human resource, and inadequate access to emergency obstetric care. The aim of this study was to determine the frequency/rate of stillbirths and its associated socio-demographic and medical factors in two rural referral hospitals in The Gambia. To our knowledge no previous study has assessed the frequency of stillbirths in The Gambia.

## 2. Materials and Methods

### 2.1. Study Setting and Design

A cross-sectional retrospective study was carried out at Bansang General Hospital and Armed Forces Provisional Ruling Council (AFPRC) Hospital. These hospitals are located in the North and South bank of The Gambia, respectively, in two different health regions. Comprehensive EOC is available most of the time, mainly provided by Cuban Medical doctors. There is no Gambian medical doctor or obstetrician in any of these hospitals. The first class of medical doctors educated in The Gambia completed their education in 2007. The two hospitals serve a population of nearly 600,000 and are referral points for nearly 30 peripheral health centres and or dispensaries. Basic EOC is not available at any of these peripheral health centres. Thus, women in either the North Bank or South Bank with obstetric complications are referred to Bansang or AFPRC hospitals. Most of these women are referred during labour. The Government of The Gambia has adopted the primary health care (PHC) strategy to make health care more accessible to the rural population. Villages with more than 400 inhabitants have resident traditional birth attendants (TBA) who have eight weeks formal training in antenatal, intrapartum and postpartum care of the mother and child. These TBAs are being supervised by a community health nurse (CHN) who is in charge of a cluster of villages. Antenatal care is provided by mobile reproductive and child health clinics from the health centres and the two hospitals.

### 2.2. Study Population

We used data from hospital records on all women who gave birth at or after 28 completed weeks of gestation from 1st July 2008 to 31st December 2008. Data was abstracted from maternity case notes, admission, and delivery registers. Midwives or doctors attending a birth complete a standardised form to be filled in upon admission and immediately after delivery. The form contains important information about maternal health and complications during pregnancy and the intrapartum period. It also contains information about the newborn. A precoded case abstraction questionnaire was used. Data abstraction was done by the principal investigator and assisted by research assistants, mainly midwives.

### 2.3. Variables

The main outcome measure was stillbirth rate calculated as deaths per 1000 births. The eligibility criteria was based on the World Health Organization's (WHOs) international comparison of viability; that is birth weight of ≥1000 g and or born at ≥28 weeks of gestation. Thus, we defined stillbirth as death in the uterus of an infant at ≥28 weeks of gestation or ≥1000 g. It was classified as fresh when the baby was born with an intact skin suggesting that the death occurred during labour (less than 12 hours before delivery). A macerated stillbirth was defined when there was sign of degeneration suggesting the death having occurred more than 12–24 hours before labour. We also recorded early neonatal deaths defined as death of the newborn baby within the first twelve hours of birth. During this period all the live born babies and their mothers were still under observation in the hospital. Due to early discharge from hospital (twelve hours after delivery) and a retrospective study design, information on live-births who might have died at home within seven days of birth was not known. Due to low number and great uncertainty these results were not included in the tables. 

For each birth, demographic and obstetric explanatory factors were captured. The demographic variables included maternal age in years, categorized in three groups: <20 (reference), 20–34, and ≥35. Parity coded as primiparous (0), 1–3 previous deliveries, ≥4 previous deliveries, area of residence (PHC village or non-PHC village). Obstetric factors included the following: admission status of the mother (booked or referred), antenatal care attendance for present pregnancy coded as no or yes, mode of delivery (spontaneous vaginal, assisted vaginal (breech), and caesarean section), presence of severe obstetric complication which included one or more of the following: prelabour rupture of membranes preterm, hypertensive pregnancy disorders (pre-eclampsia and eclampsia), antepartum haemorrhage comprising (placenta previa and abruption placenta), cephalopelvic disproportion (CPD), prolonged or obstructed labour, severe anaemia (haemoglobin level <9 g/dL). Foetal characteristic was birth weight <2500 g and ≥2500 g. 

A total of 1,849 maternity admissions were recorded during the six months period. We excluded 224 (12.2%) who had not delivered. Twenty-one births were further excluded due to missing information on the vital status and birth weight, and 25 who weighed less than 1000 g. We also excluded 60 deliveries that occurred before reaching the hospital. The final data set for this analysis was 1,519. The Ethics Committee of Norway and the Joint Gambia Government and Medical Research Council Review board approved the study. Permission to carry out the study was achieved from the Ministry of Health of The Gambia and the chief executive officers of Bansang and AFPRC hospitals.

### 2.4. Statistical Analysis

All 1,519 institutional births were included in the analysis. Frequency analysis and cross-tabulations were used to determine the frequency and percentage of stillbirth and early neonatal mortality. Overall, stillbirth was calculated as a proportion of all births while early neonatal mortality was presented as a proportion of live births. Fresh and macerated stillbirths were calculated as a proportion of stillbirths. Multiple births were initially excluded, but repeating the analysis including multiple births the stillbirth rate remained largely unchanged. Thus we decided to maintain multiple births in the analyses. Univariate association between covariates and stillbirths were assessed with chi-square test or Fisher's exact test as appropriate. All *P*-values were two-sided and a value of 0.05 was considered statistically significant. Finally all covariates were included in a multivariate logistic regression model to determine significant factor associated with stillbirth. All statistical analysis was done with Software Package for Social Sciences (SPSS) for Windows version 16.0 (SPSS Inc, Chicago, II, USA).

## 3. Results

The total number of deliveries over the six months period was 1,519. Of these, 237 were stillbirths, representing a stillbirth rate of 156 (95% CI 138–174) per 1000 births. Of the 237 recorded stillbirths, 137 (57.8%) were fresh stillbirth. We recorded 6 early hospital neonatal deaths giving a hospital neonatal mortality rate of 5 (95% CI 2–10) per 1000.

More than half (54.9%) of the women were between 20–34 years old. Forty-six percent were primiparous. Nearly all (99.3%) attended antenatal care at least once. Most of the women (89%) had a spontaneous vaginal delivery, 8% delivered by caesarean section, while 3.4% of the births were assisted breech delivery. Twenty-two percent of the women were referred from peripheral health centres. Of the 1,519 recorded deliveries the partograph was not used in 958 (63%). Overall, 53 (3.5%) of the women had a breech presentation at delivery (Table 1).

Table 1 shows the results of the univariate analysis. The crude analysis indicate that stillbirths were highly significantly associated with the following factors: referral from a peripheral health facility (odds ratio: 13.75, 95% CI (10.02–18.84), severe obstetric complication 11.74, 95% CI (8.58–16.06), birth weight <2500 g 6.32, 95% CI (4.67–8.55), residence in a PHC village 2.13, 95% CI (1.59–2.80), nonuse of patograph 2.48, 95% CI (1.78–3.45) and ≥4 pregnancies 2.24, 95% CI (1.61–3.10). Lack of antenatal care, assisted breech delivery, and maternal age 35 years and above were also associated with high stillbirth rate, OR = 5.5, 95% CI (1.58–19.16), 1.80, 95% CI (1.16–2.80), and 1.80, 95% CI (1.14–2.83), respectively. After adjusting for all the variables in a multivariate logistics analysis, presence of severe obstetric complication(s), birth weight <2500 g, caesarean section delivery and referral from a peripheral health facility were the most important factors highly significantly associated with higher stillbirth rates OR = 6.68 (95% CI. 3.84–11.62), 4.47 (95% CI. 3.04–6.59), 4.35 (95% CI 2.46–7.69), and 3.82 (95% CI. 2.24–6.51), respectively. However, the proportion of stillbirths was relatively lower in elective c/s groups (20.8%) than for the emergency c/s groups (23.5%), *P* > .05. In addition, other factors associated with high stillbirth rate were nonuse of partograph OR = 1.70 (95% CI 1.23–2.56), multiple pregnancy OR = 2.01 (95% CI 1.05–3.86), and not attending antenatal care OR = 4.46 (95% CI 0.84–23.43) (Table 1). 

The association between maternal demographic/obstetric factors and fresh stillbirths are presented in Table 2. On univariate analysis, complications during intrapartum period, being delivered at AFPRC hospital and birth weight <2500 g were significantly associated with fresh stillbirth, OR = 3.57 (95% CI 1.52–8.40), 2.15 (95% CI 1.26–3.66), and 2.15 (95% CI 1.27–3.63), respectively. After adjusting for the effect of all the variables in a multivariate analysis intrapartum severe obstetric complication was the only independent factor associated with high rate of fresh stillbirth; OR = 3.14, 95% CI (1.01–9.76).

Of the 1339 live births registered during the study period, 11 maternal deaths were recorded representing a hospital maternal mortality rate of 822/100,000 live births (LB). Of the 11 recorded maternal deaths, 7 (1,169/100,000 LB) and 4 (541/100,000 LB) were in Bansang and AFPRC hospitals, respectively.

## 4. Discussions

### 4.1. Main Results

The stillbirth rate found in the two rural hospitals in The Gambia was unacceptably high, pegging at 156 per 1000 total births. The reported early neonatal deaths rate was 5/1000 live births. Presence of severe obstetric complication showed a close association with stillbirth, followed by low birth weight, caesarean section, and referral from a peripheral health facility. Stillbirth was also associated with nonused of the partograph, multiple pregnancy, and lack of antenatal care. In addition, obstetric complications during the intrapartum period were independently associated with fresh stillbirths.

### 4.2. Methodological Considerations

Even though hospital-based data has a limitation in the correct appraisal of the magnitude of a problem in the general population, lack of nationwide vital registration system in many developing countries including The Gambia, has made population-based studies unfeasible. Some field reports on stillbirths from Zimbabwe [7] and The Gambia [9] were established from hospital data. These data are however very vital in rendering both clinical and research priorities. Several limitations of this study should be recognized. Due to the hospital-based design of the current study, we might have overestimated the stillbirth rate. The reported numbers of early neonatal mortality are small and such deaths may also underestimate the true neonatal mortality rates since no systemic follow-up mothers or infants were undertaken. Some of the women may have experienced neonatal deaths after discharge from hospital. Due to the practice of hospital discharge within 12 hours after delivery most of the early neonatal deaths were also not captured in the maternity records. Usually hospital data will show a very high percentage of deaths due to asphyxia since complicated births are more likely to come to hospital. The very small number of observations of early neonatal deaths in our study gives estimates with large uncertainty. Thus, the findings of our study cannot be generalized to the entire country and should be interpreted with caution.

### 4.3. Stillbirth Rates and Associated Factors

The reported stillbirth rate in the current study is higher than in a recent hospital-based study by Cham et al., 116 per 1000 births [9]. The rate is also higher than in other previously reported findings from The Gambia [10, 11] and, in one hospital-based study conducted in Zimbabwe, 61 per 1000 births [12]. Our rate is also considerably increased compared to the reported rates within Sub-Saharan Africa; 32.2 per 1000 births [5], and the WHO model estimates of 42 per 1000 births [2]. We speculate that the higher rates registered in our study could be attributed to the referral of complicated obstetric cases from peripheral health centres. Most of the obstetric cases referred often reach the hospital when it is already late. In addition, the high stillbirth rate may be in part due to the low degree of obstetric vigilance and improper labour management. Our study demonstrated a very high stillbirth rate and a relatively very low neonatal mortality rate. This may indicate a serious delay on behalf of the baby. Thus, it would be reasonable to assume that the babies do not even live to be born “asphyxiated.” However, the small number of neonatal deaths gives insufficient power to conclude on this matter. 

Unavailability and high cost of transportation, poor road conditions, and time to arrange for transport from remote villages may increase the time to reach a health facility [13]. Such factors could play an important role to the findings of the current study. At the time of an obstetric emergency, every moment of delay in seeking and receiving skilled care increases the risk of stillbirth, neonatal or maternal death or disability. If there were fewer delays, fewer babies would probably be stillborn, but many more would be born asphyxiated or die early. Reducing transport time to an EOC facility is challenging in rural settings where roads, public transportation, and communication infrastructures are poor and the terrain may be formidable [13]. Evidence exists that a functioning continuum of care between the home, health centre, and hospital is required to minimize potentially deadly delays and effectively link pregnant women and newborns to skilled obstetric and newborn care [13]. However, even where prompt referral was initiated, the gross shortage of trained human resources for health and inadequate facilities for emergency obstetric and neonatal care must be overcome to reduce perinatal deaths in rural hospitals in The Gambia. 

Traditionally, advanced maternal age is viewed as risk factor for pregnancy complications and adverse perinatal outcomes including stillbirths [14, 15]. However, the biological mechanism underlying the increased risk for adverse perinatal outcomes with advanced maternal age is unclear [15]. Older mothers in our population had the highest percentage of stillbirths and the proportion of stillbirth increased steadily as age increases. However, the significant association was lost after adjusting for all the variables included in the study. 

Almost all the women in our study (99%) attended antenatal care at least once. However, the percentage of stillbirth was much higher among mothers who did not attend antenatal care compared with those who did. This is consistent with results of other studies carried out elsewhere [12, 16]. Better understanding of foetal growth and development and its relationship to the mother's health has resulted in increased attention to the potential of antenatal care as an intervention to improve both maternal and newborn health [17]. Antenatal care provides a critical linkage between the woman and maternity care services. Thus, if promoted in concurrence with effective EOC and delivered in skilled hands, it may become an effective instrument to improve maternal and perinatal birth outcomes particularly in developing countries [18]. However, opponents maintained that antenatal care could only detect morbidity during pregnancy and could not detect obstetric complications that would occur during labour [19]. 

Noncompliance in completing the partograph is common in Gambian hospitals. A higher percentage of stillbirths were observed in situations where the partograph was not used. In a recent paper by Cham et al. on foetal outcome in severe maternal morbidity, the partograph was not used in any of the 725 identified hospital deliveries [9]. The partogram graphically represents key events during labour. It is recommended for routine monitoring of labour to provide an early warning system; thus, assists the health worker to identify slow progress in early labour, hence, initiate appropriate interventions to avert prolonged and obstructed labour [20]. Few studies have assessed partograph used versus no partograph, the impact of which would be underestimated in higher-resourced settings where all women have close surveillance by experienced clinicians. In a larger WHO prospective study in South East Asian Hospitals, partograph used was found to be associated with reduced prolonged labour and stillbirth [21]. Inadequate intrapartum foetal monitoring may result in untimely execution of life-saving intervention, particularly in complicated deliveries. Thus, in low-resource settings partograph use is recommended for monitoring all women in labour, and can serve as a guide for timely referral to Comprehensive Emergency Obstetric Care (CEmOC) facilities [22]. 

Complications during pregnancy and childbirth have been long known to increase the risk of perinatal death. In our study a higher percentage of women admitted with obstetric complications lost their babies either during pregnancy, labour or shortly after delivery. This is consistent with findings by Cham et al. 2009 [9]. The high rate of stillbirth in multiple pregnancies and deliveries complicated by breech presentation in our study reaffirmed the call that screening for abnormal foetal presentations and multiple pregnancies could be one key component of antenatal care particularly in developing countries. Vanneste et al. demonstrated in one community-based study in Bangladesh that measurement of the fundal height had availed midwives the opportunity to identify a large proportion of women with twin pregnancies [18]. We speculate that timely referral of breech and twin pregnancies to an EOC facility will avert some of the adverse birth outcomes. Thus, refresher training in breech and twin delivery coupled with the presence of a skilled attended at all such deliveries seems warranted.

Most of the stillbirths in our study were fresh, with a fresh to macerated stillbirths ratio of 1.3 : 1. This indicates that most of the deaths probably occurred during labour, Fresh stillbirths are often used as proxy for stillbirths due to acute intrapartum insults [23]. The ratios of fresh to macerated stillbirths may indicate the availability and quality of prenatal and obstetric care, with high ratios suggesting inadequate or poor quality of EOC [12]. Thus, our results reaffirm the need for improved and timely access to EOC services during the intrapartum period in rural Gambia. In developed countries, less than 10% of all stillbirths are intrapartum stillbirths, while in developing countries almost one-half of all stillbirths are assumed to be intrapartum related [23]. The rate of intrapartum stillbirths have been substantially reduced in developed countries. This significant decline is mainly due to increased availability, quality, and access to EOC services and better intrapartum monitoring of high risk births [24]. Thus, understanding the burden of stillbirth has important programmatic and resource implications, which are of special interest in very low-resource settings like rural Gambia [25].

The unmet need for obstetric care is high in developing countries where most of the intrapartum stillbirths take place [26]. However, many of these deaths could be prevented with improved obstetric care [27]. The relatively low rate of intrapartum stillbirth in developed countries was to a larger extent due to the timely purveying of caesarean section [28]. Additionally, in developing countries caesarean section availability was associated with diminution in intrapartum stillbirth rates [27, 28]. However, nonavailability of EmOC, particularly caesarean section has been implicated as a risk factor for intrapartum stillbirth especially in cases of prolonged labour [29]. While caesarean section can be a life saving interposition for mother and child, evidence showed that its use, particularly in low-resource settings could be associated with increased risk of perinatal mortality, especially when it is performed late [30]. Consistent with the above findings, caesarean section deliveries in our study showed a higher proportion of fresh stillbirths when compared with a normal vaginal delivery. In addition, a larger proportion of stillbirths are observed in emergency c/s groups when compared with elective c/s group. We therefore speculate that caesarean sections are probably applied too late in these hospitals resulting to not saving the baby's life. This emphasises the importance of performing caesarean section (C/S) with the correct timing because early c/s could save more lives. Therefore, the increased risk of stillbirths must be considered when c/s is performed in the late stage of labour. The availability of quality EOC, pendent by emergency transport services and skilled providers is pivotal for effective maternal health services in The Gambia.

## 5. Conclusions

Our findings suggest that the stillbirth rate is unacceptably high in rural hospitals in The Gambia. The findings also reaffirmed the important contribution of severe obstetric complications on the birth outcome in these settings. We also demonstrated an association between stillbirths and nonuse of the partograph, as well as with assisted breech and caesarean section. Most of the stillbirths were fresh, suggesting that these deaths have occurred during labour or shortly before delivery, thus potentially avertable. Improved intrapartum care through safe, comprehensive essential, and emergency obstetric supported by emergency transport services and skilled personnel is warranted for improved foetal out-comes in low resource settings such as The Gambia.

##  Competing Interest

No competing interest. The authors are responsible for the content and writing of this paper.

## Figures and Tables

**Table 1:** Demographic/reproductive and obstetric factors associated with stillbirth.

Characteristics	Total birth *n* (%)	Stillbirth *n* (%)	Crude OR (95% CI)	Adjusted^aa^ OR (95% CI)
	**1519**	**237(15.6)**		
Maternal age (yrs)				

<20	509(33.5)	61(12.0)	1	1.
20–34	827(54.4)	140(16.9)	1.20(0.80–1.81)	0.97(0.57–1.65)
≥35	183(12.0)	36(19.7)	1.80(1.14–2.83)*	1.39(0.64–2.93)

Parity				

0	703(46.3)	77(11.0)	1	1.
1–3	380(25.0)	66(17.4)	1.31(0.92–1.86)	0.48(0.28–0.78)
≥4	436(28.7)	94(21.6)	2.24(1.61–3.10)***	0.35(0.20–0.63)

Residence				

PHC Village	473(31.1)	108(22.8)	2.13(1.59–2.80)***	1.14(0.78–1.66)
Non-PHC Village	1046(68.9)	129(12.3)	1	.

Admission status				

Referred	303(19.9)	153(50.5)	13.75(10.02–18.84)***	3.82(2.24–6.51)***
Booked	1216(80.1)	84(6.9)	1	1.

Antenatal Care				

No	10(0.7)	5(50.0)	5.50(1.58–19.16)**	4.45(0.84–23.43)
Yes	1509(99.3)	232(15.4)	1	1.

Mode of delivery				

Spontaneous Vaginal	1340(88.2)	191(14.3)	1	1
Assisted breech	53(3.5)	17(32.1)	1.80(1.16–2.80)**	1.64(0.74–3.60)
Caesarean Section	126(8.3)	29(23.0)	0.63(0.31–1.29)	4.35(2.46–7.70)***

Used of Patograph				

No	958(63.1)	187(19.5)	2.48(1.78–3.45)***	1.70(1.13–2.56)**
Yes	561(36.9)	50(8.9)	1	1.

Obstetric complication				

Yes	370	164(44.3)	11.74(8.58–16.06)***	6.68(3.84–11.62)***
No	1149	73(6.4)	1	1.

Type of birth				

Multiple	111(7.3)	20(18.0)	1.20(0.73–1.99)	2.01(1.05–3.86)**
Singleton	1408(92.7)	217(15.4)	1	1.

Birth weight				

<2500 g	278(18.3)	114(41.0)	6.32(4.67–8.55)***	4.48(3.04–6.59)***
≥2500 g	1241(81.7)	123(9.9)	1

^aa^Adjusted for all variables listed in the table. *P-value <.01, **P-value <.05, ***P-value <.001.

**Table 2:** Maternal demographic and obstetric factors associated with fresh stillbirth (FSB) (%).

Profile	FSB (%)	Crude OR (95% CI)	Adjusted^aa^ OR (95% CI)
Age (years)			

<20	54.1	0.72(0.39–1.32)	0.76(0.27–2.15)
20–34	62.1	1.32(0.58–3.01)	1.91(0.42–8.41)
≥35	47.2	1	1

Parity			

0	59.7	1.24(0.64–2.40)	1.08(0.37–3.12)
1–3	54.5	1	1
≥4	58.5	1.05(0.57–1.94)	0.48(0.15–1.48)

Recruiting hospital			

Bansang	49.6	1	1
AFPRC	67.9	2.15(1.26–3.66)**	1.42(0.66–3.01)

Residence			

PHC Village	56.5	1	1
Non-PHC Village	58.9	1.11(0.66–1.85)	0.78(0.38–1.58)

Antepartum admission			

Yes	52.8	0.65(0.39–1.10)	0.80(0.30–2.10)
No	63.2	1	1

Timing of complication			

Antepartum	50.4	1	1
Intrapartum	78.4	3.57(1.52–8.40)**	3.14(1.01–9.76)**

Partograph used			

No	58.3	1.10(0.59–2.06)	1.73(0.70–4.30)
Yes	56.0	1	1

Mode of delivery			

Spontaneous vaginalBreech/others	55.5	1	1
Breech/others	64.7	1.43(0.40–5.18)	1.23(0.42–3.57
Caesarean Section	72.4	2.15(0.91–5.11)	1.32(0.32–5.45)

Birth weight (grms)			

<2500	48.2	1	
≥2500	67.7	2.15(1.27–3.63)**	1.67(0.81–3.44)

^aa^Adjusted for all variables listed in the table. **P-value <.05.

## Abbreviations

MDGs: Millennium development goals
EOC: Emergency obstetric care
AFPRC: Armed forces provisional ruling council
PHC: Primary health care
TBA: Traditional birth attendant
ENNM: Early neonatal neonatal mortality
CEmOC: Comprehensive emergency obstetric care.

## References

[1] Kramer MS (2003). The epidemiology of adverse pregnancy outcomes: an overview. *Journal of Nutrition*.

[2] World Health Organization (2006). *Neonatal and Perinatal Mortality. Country, Regional and Global Estimates*.

[3] Kusiako T, Ronsmans C, van der Paal L (2000). Perinatal mortality attributable to complications of childbirth in Matlab, Bangladesh. *Bulletin of the World Health Organization*.

[4] Kambarami RA (2002). Levels and risk factors for mortality in infants with birth weights between 500 and 1,800 grams in a developing country: a hospital based study. *Central African Journal of Medicine*.

[5] Stanton C, Lawn JE, Rahman H, Wilczynska-Ketende K, Hill K (2006). Stillbirth rates: delivering estimates in 190 countries. *The Lancet*.

[6] Osman NB, Challis K, Cotiro M, Nordahl G, Berström S (2001). Perinatal outcome in an obstetric cohort of Mozambican women. *Journal of Tropical Pediatrics*.

[7] Feresu SA, Harlow SD, Welch K, Gillespie BW (2004). Incidence of and socio-demographic risk factors for stillbirth, preterm birth and low birthweight among Zimbabwean women. *Paediatric and Perinatal Epidemiology*.

[8] The Gambia Government (2005). *The Gambia Road Map to Accelerate the Reduction of Maternal and Newborn Morbidity and Mortality*.

[9] Cham M, Sundby J, Vangen S (2009). Fetal outcome in severe maternal morbidity: too many stillbirths. *Acta Obstetricia et Gynecologica Scandinavica*.

[10] Department of State for Health (2002). *Report on the National Survey on Maternal, Perinatal, Neonatal, and Infant Mortality and Contraceptive Prevalence*.

[11] Greenwood AM, Greenwood BM, Bradley AK (1987). A prospective survey of the outcome of pregnancy in a rural area of the Gambia. *Bulletin of the World Health Organization*.

[12] Feresu SA, Harlow SD, Welch K, Gillespie BW (2005). Incidence of stillbirth and perinatal mortality and their associated factors among women delivering at Harare Maternity Hospital, Zimbabwe: a cross-sectional retrospective analysis. *BMC Pregnancy and Childbirth*.

[13] Lee AC, Lawn JE, Cousens S (2009). Linking families and facilities for care at birth: what works to avert intrapartum-related deaths?. *International Journal of Gynecology and Obstetrics*.

[14] Berkowitz GS, Skovron ML, Lapinski RH, Berkowitz RL (1990). Delayed childbearing and the outcome of pregnancy. *The New England Journal of Medicine*.

[15] Hsieh T-T, Liou J-D, Hsu J-J, Lo L-M, Chen S-F, Hung T-H (2010). Advanced maternal age and adverse perinatal outcomes in an Asian population. *European Journal of Obstetrics Gynecology and Reproductive Biology*.

[16] Galvin J, Woelk GB, Mahomed K, Wagner N, Mudzamari S, Williams MA (2000). Perinatal care utilization and foetal outcomes at Harare maternity hospital, Zimbabwe. *Central African Journal of Medicine*.

[17] Making Pregnancy Safer, World Health Organization (2003). *Antenatal Care in Developing Countries, Achievements and Missed Opportunities: An Analysis of Trends, Levels and Differentials, 1990–2001*.

[18] Vanneste AM, Ronsmans C, Chakraborty J, De Francisco A (2000). Prenatal screening in rural Bangladesh: from prediction to care. *Health Policy and Planning*.

[19] Fahdhy M, Chongsuvivatwong V (2005). Evaluation of World Health Organization partograph implementation by midwives for maternity home birth in Medan, Indonesia. *Midwifery*.

[20] World Health Organization (2006). *Pregnancy, Childbirth, Postpartum and Newborn Care: A Guide for Essential Practice*.

[21] Kwast BE (1994). World health organization partograph in management of labour. World Health Organization Maternal Health and Safe Motherhood Programme. *The Lancet*.

[22] Hofmeyr GJ, Haws RA, Bergström S (2009). Obstetric care in low-resource settings: what, who, and how to overcome challenges to scale up?. *International Journal of Gynecology and Obstetrics*.

[23] Lawn J, Shibuya K, Stein C (2005). No cry at birth: global estimates of intrapartum stillbirths and intrapartum-related neonatal deaths. *Bulletin of the World Health Organization*.

[24] Goldenberg RL, Kirby R, Culhane JF (2004). Stillbirth: a review. *Journal of Maternal-Fetal and Neonatal Medicine*.

[25] McClure EM, Wright LL, Goldenberg RL (2007). The global network: a prospective study of stillbirths in developing countries. *American Journal of Obstetrics and Gynecology*.

[26] Prytherch H, Massawe S, Kuelker R, Hunger C, Mtatifikolo F, Jahn A (2007). The unmet need for emergency obstetric care in Tanga Region, Tanzania. *BMC Pregnancy and Childbirth*.

[27] McClure EM, Goldenberg RL, Bann CM (2007). Maternal mortality, stillbirth and measures of obstetric care in developing and developed countries. *International Journal of Gynecology and Obstetrics*.

[28] Goldenberg RL, McClure EM, Bann CM (2007). The relationship of intrapartum and antepartum stillbirth rates to measures of obstetric care in developed and developing countries. *Acta Obstetricia et Gynecologica Scandinavica*.

[29] Mooney SE, Ogrinc G, Steadman W (2007). Improving emergency caesarean delivery response times at a rural community hospital. *Quality and Safety in Health Care*.

[30] De Muylder X, Amy J-J (1993). Caesarean section rates in an African country. *Paediatric and Perinatal Epidemiology*.

